# The Correlation between Cervical Fusion Length and Functional Outcomes in Patients with Traumatic Spinal Cord Damage—A Registry-Based Cohort Study

**DOI:** 10.3390/jcm11195867

**Published:** 2022-10-04

**Authors:** Yannick Rau, Roland Thietje, Arndt-Peter Schulz, Marc Auerswald, Ralf Böthig, Sven Hirschfeld

**Affiliations:** 1 Faculty of Medicine, Universität zu Lübeck, 23562 Lübeck, Germany; 2 Spinal-Cord-Injury Center, BG Klinikum Hamburg, 21033 Hamburg, Germany; 3 Zentrum Klinische Forschung, BG Klinikum Hamburg, 21033 Hamburg, Germany; 4 Department of Trauma and Orthopaedic Surgery, BG Klinikum Hamburg, 21033 Hamburg, Germany

**Keywords:** traumatic spinal cord injury, cervical spine, interbody fusion, rehabilitation

## Abstract

This study aims to assess if there is an evident correlation between fusion length and rehabilitation success after trauma to the cervical spine that could potentially be used to predict functional outcomes. This monocentric study was conducted in the Spinal-Cord-Injury center of the Berufsgenossenschaftliches Klinikum Hamburg. Data sets of 199 patients from the Spinal-Cord-Injury center admitted between the beginning of 2003 and the end of 2018 were subjected to statistical analyses. The Spinal Cord Independence Measure II (SCIM II) difference between admission and discharge was chosen as the primary outcome variable of a multiple linear regression analysis, including several other variables. The length of fusion, SCIM at admission and the International Standards for Neurological Classification of Spinal Cord Injury (ISNCSCI) values at admission could be identified as significant predictors. The cervical fusion length could be identified as an independent predictor of the functional outcome within our model. This correlation most likely mediates for the range of motion as well as partly for injury severity. This is much harder to evaluate in a newly admitted rehabilitation patient than a single numerical value that represents its rehabilitative implications, such as fusion length. Together with an initial assessment of the SCIM and ISNCSCI, it provides a solid basis for outcome prediction.

## 1. Introduction

Research data regarding the epidemiological details of individuals with cervical trauma and their associated spinal cord injury are sparse. A meta-analysis by Milby et al. in 2008 suggested that approximately 3.7% of all trauma patients suffer from a cervical spine injury. Approximately 42% of those injuries are unstable injuries, and patients are therefore at risk of developing spinal cord injury (SCI) [1]. An analysis by Stephan et al. from 2015 led us to conclude that injuries to the cervical spine are the most frequent causes of traumatic spinal cord injury in Germany and can be calculated at an incidence of approximately 202 individuals per 82 million a year [2].

Cervical spinal injuries, if unstable, are commonly treated surgically. Vertebral bodies are stabilized via osteosynthesis and interbody fusion [3,4,5,6,7]. Although major progress within this area of spinal surgery has been achieved to reduce the perioperative complications and revision rates, the method of vertebral fusion and fixation retains its inherent compromise between the stability and reduced mobility of the spine. While the surgical approach (anterior, posterior, and combined) and the used fusion system (e.g., cage and bone graft) are different, the ultimate result remains spondylodesis, with fused vertebrae that are completely restricted in mobility. This becomes more and more important during the later stages of rehabilitation. In rehabilitative practice, it is well known that patients with plegia or paresis below the cervical level are highly reliant on mobility to perform daily tasks. This includes, but is not limited to, personal hygiene, tool usage, and eating.

The current literature mainly addresses the immediate success of surgery as one of the main interests. However, it may be beneficial also to take rehabilitation success into account and identify the potential predictors of it. This appears to be one of the most underdeveloped areas of research regarding spinal cord injury.

To change this, a look at the clinical documentation and patients’ histories is necessary. Several clinical tools are required.

The documented extent of a spinal cord injury and the subsequent impairment is largely determined by its clinical presentation. To quantify and categorize this, the American Spinal Cord Injury Association (ASIA) published a widely used instrument, the International Standards for Neurological Classification of Spinal Cord Injury (ISNCSCI), or the ASIA Impairment Scale. It can be used to determine the sensory and motor function of individuals with spinal cord injuries [8]. The ISNCSCI was shown to be satisfyingly reliable and valid in several studies and can be used to determine an individual’s prognosis and neuromotorical status [9,10,11,12].

To further evaluate the functionality and its development, other scores are required to provide an in-depth view of the skills necessary to perform daily tasks. The 2001 Catz-Itzkovich Spinal Cord Independence Measure II (SCIM II) is such a score [13,14]. It was shown to be reliable and valid while simultaneously being more sensitive to changes than other means of measurement, such as the Functional Independence Measure (FIM) [15,16].

Residual functionality is of utmost importance to individuals suffering from spinal cord injury and even more in those with an injury to the cervical spine. Therefore, it is essential to preserve as much of it as possible. This also includes mobility, especially in those who suffer from severe cervical spinal cord injury and are heavily reliant on head movements. Though it may seem plausible, it has not yet been proven whether the number of artificially connected vertebral bodies correlates with the participant’s rehabilitative outcome in any meaningful way and if fusion length can be used to predict rehabilitative success. The length of fusion could then be used as an additional tool to establish scoring instruments, such as the ISNCSCI and SCIM, to further determine a patient’s prognosis and rehabilitation needs. 

## 2. Materials and Methods

**Figure 1:**
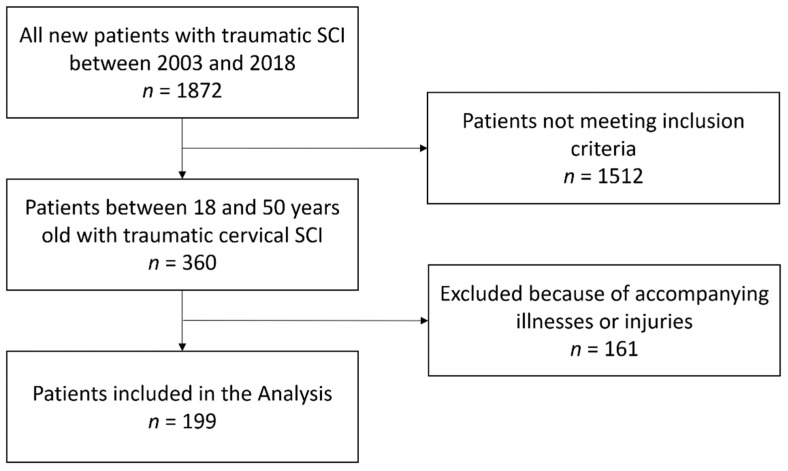
Inclusion and exclusion criteria before analysis. SCI = Spinal Cord Injury.

The inclusion criteria for this study were as follows: Participants between the ages of 18 and 50 who suffered from traumatic cervical spine injuries with additional cervical spinal cord damage. Patients were treated with a vertebral fusion of at least one cervical segment and began their rehabilitation at the Spinal-Cord-Injury center in the Berufsgenossenschaftliches Klinikum Hamburg during the period of 2003 to the end of 2018. We limited the age to 50 years to combat the confounding factors that are mainly influenced by age-related functionality reductions and reduce rehabilitation success. Fifty years was an established cutoff in previous publications regarding rehabilitation success in individuals with spinal cord injuries [17,18,19]. A total of 360 individuals could be identified to fit these criteria. Individuals with significant additional illnesses or injuries impairing their functionality, as well as persons with consuming illnesses, were excluded from the analysis. This includes, but is not limited to, individuals suffering from cancerous lesions of the neurological, skeletal, or muscular system, degenerative diseases including spinal stenosis, individuals suffering from severe hemorrhages to the brain or brainstem, as well as individuals suffering from apoplectic strokes. Persons with amputations or severe irreparable lesions of the peripheral nervous system, such as plexus injuries, were also excluded. The enrollment is also presented in Figure 1.

The following data were recorded:

Age at injury in years;

Gender;

Surgical approach (anterior vs. posterior or combined);

Time from injury to admission to the rehabilitation center in days;

Length of stay in days;

Spinal cord level of injury;

Length of interbody fusion;

ISNCSCI values and grades at admission; 

The Spinal Cord Independence Measure II (SCIM II) at admission and discharge.

The SCIM version II was chosen as version III was only finally validated in 2007, and we assumed better comparability between individuals when using a consistent instrument [20].

The ISNCSCI provides two possible uses. It can be used to determine the injury severity by assigning a letter from A to E to each patient. This can be used to quickly determine if an injury is complete or incomplete and how severely the impairment affects the patient. This classification of AIS types is mainly used for descriptive purposes and is used in this study accordingly. For statistical analysis, the AIS score, also commonly referred to as the ISNCSCI score, provides a numerical score for sensory and motor functions. This continuous variable is used in our analysis as it provides more in-depth details on the patient’s neurological state and the severity of the injury.

The length of fusion was determined using the surgery reports submitted by primary care facilities to the Spinal-Cord-Injury center at patient admittance. The radiological evaluation of injury was performed in those primary care facilities prior to surgery and always included three-dimensional imaging.

For the statistical analysis, Jamovi (version 1.2.2.0, The jamovi project, Sydney, Australia), an R-based tool, was used.

The mean and standard deviation (SD) were supplied if possible. The frequencies or percentages were added if the values were categorical.

In the case of the SCIM II values, we conducted a multiple linear regression analysis on the values of the difference between admission and discharge, assuming a continuous scale of SCIM values as was conducted in previous publications [13,14,15,16]. We identified the following variables as those of interest and possible explanatory variables:

Age at injury (in years);

Time until admission (in days);

Level of neurological injury at admission;

Length of interbody fusion;

Total ISNCSCI value at admission;

SCIM II at admission.

To include the spinal cord level of injury, anatomical levels between C0 and C8 were translated into a numerical scale of 0 to 8. An interval scale measurement was assumed.

The usage of a multiple linear regression model rather than regular parametric tests and the inclusion of these criteria serve the purpose of retrospectively minimizing the most probable confounding factors. 

The regression coefficients and their respective *p* values, as well as the 95% confidence intervals, are presented. Coefficients were considered to deviate significantly from 0 at an α level of 0.05.

In addition, we calculated the variance inflation factors (VIFs) to exclude multicollinearity prior to the analysis and presented the F-Tests to verify the significance of the predictive value of our model. VIF values below 10 were considered to be acceptable. To ensure that the linearity assumptions were met, the model fitness was quantified using the coefficient of determination of Nagelkerke’s R, stated as R^2^ and adjusted R^2^. The study protocol was presented to the ethical committee of the Universität zu Lübeck, which granted permission for the study (reference number 20-336).

## 3. Results

A total of 199 individuals were identified to fit the inclusion criteria for this study. A total of 170 (85.4%) were male. The mean (SD) age was 31.5 (9.82) years.

The mean (SD) time until admission to the spinal rehabilitation unit was 11.6 (14.5) days after trauma. The length of stay was documented in 192 cases, and the mean (SD) was determined to be 169 (81.4) days. At the time of admission (T1), 49.7% suffered from a complete injury (AIS Type A). At the time of discharge (T2), this number decreased to 39.2%. There were 127 individuals (63.8%) from C0 to C4 and 72 individuals (36.2%) from C5 to C8. A more detailed account of the demographic details can be observed in Table 1.

The mean (SD) interbody fusion length for all patients was determined at 3.16 (1.24) vertebral bodies. A detailed description of the fusion length and neurological level of injury can be seen in Table 2. 

The anterior surgical approach was the most common in 156 individuals (78.4%). Ten individuals were treated with posterior fusion, and 33 individuals were subject to a combined or 360-degree method. Four participants also received a surgical fixation of their atlas or axis, and 34 fusions included the vertebral bodies of the thoracic spine and therefore crossed the cervicothoracic junction. All fusions needed to include the cervical spine, at least partially. The mean (SD) fusion length of the anterior fusion was 2.87 (0.78), while the combined fusion was 4.42 (1.92), and the posterior fusion was 3.5 (1.6). Table 3 shows the upper fusion levels of the patients in relation to the surgical approach.

For all 199 participants, the ISNCSCI Motor Score values were documented at admission (T1). Almost the same applies to the sensory portion of the ISNCSCI. Here, only 198 (T1) were found (Table 4).

### Spinal Cord Independence Measure II 

The SCIM II values were collected for 198 participants at admission and 199 individuals at release. For those 198 individuals with available scores at admission and release, the differences were calculated (Table 5).

The longer the fusion length, the smaller the number of patients. This led to reduced comparability of means. For example, one patient with seven vertebrae fusions did exceptionally well by gaining 52 points in the SCIM throughout his rehabilitation. This led to an increase in the mean SCIM difference in a group of only two patients, in which the other one only gained 21 points. Both patients’ results were within the margins of error, but the comparability to other subgroups was significantly reduced. Additionally, within these subgroups, we did not yet adjust for other background factors, such as the ISNCSCI or SCIM at admission. A different analysis is required to adjust for these factors and to properly reflect a possible correlation. A mere comparison between the length groups presented would allow for too much confounding to occur.

**Figure 2:**
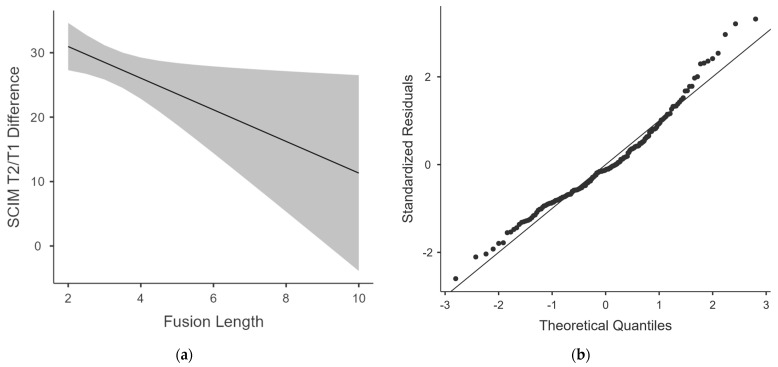
(**a**) The estimated marginal means plot of the Spinal Cord Independence Measure differences in relation to fusion length; (**b**) the Q-Q plot of the linear regression model.

For those participants that had complete data sets, including the ISNCSCI total scores at admission, a multiple linear regression was calculated (Table 6). An estimated marginal means plot is supplemented for the graphical representation of our regression model (Figure 2a). In addition, a Q-Q plot is provided to illustrate the models’ fitness (Figure 2b).

The calculated model fitness of the adjusted R^2^ = 0.29 and Q-Q plot observations led to the reasonable assumption of at least moderate linearity.

A significant correlation between the length of fusion and SCIM difference was found whilst also identifying age and total ISNCSCI values at admission as significant coefficients and potential causes of interference. The results of our calculation suggest a loss of approximately 2.4 SCIM points per added segment of fusion if all other variables are fixed. As the 95% confidence interval (95% CI, −4.6 to −0.21) had a negative upper end, a negative correlation between the length of fusion and SCIM difference is mathematically very certain.

## 4. Discussion

We conducted a cohort study on one of the largest collectives of individuals suffering from cervical spinal cord injury available regarding the number of fused segments. The main goal was to evaluate the length of vertebral fusion as a potential indicator for reduced rehabilitation success. This allowed us to reduce potential bias and mathematically streamline our study population. It also allowed us to perform multidimensional analyses. In contrast to conventional group comparisons, this analysis created a linear model with all chosen variables influencing its trajectory and created the best explaining function of the chosen outcome variable with all other variables as independent coefficients.

We included the SCIM II values at admission as well as the ISNCSCI values at admission, which are influenced by any functionally limiting condition of the patient and, therefore, can be assumed to be altered by such conditions. Both were previously shown to be predictors of rehabilitation success and have independent value in detecting functional changes and outcomes [21,22,23]. This can also be supported by our findings. Both were significant predictors of rehabilitation success. Our analysis showed that a VIF < 10 was present within our model for the ISNCSCI and SCIM at admission. This does not mean that no correlation between the two was present but indicates that in the context of our multidimensional model, both provided independent predictive values to our outcome parameter of SCIM difference. Evaluating both scores at admission to the rehabilitation center, therefore, provides independent explanatory value to the prediction of the rehabilitation outcome. 

Most notably, the length of fusion could be determined as a significant independent predictor. This shows once more that neurological outcomes and functional outcomes are not equal. Instead, the functional outcome, as represented with the SCIM, is dependent on the neurological outcome, as shown by its correlation to the ISNCSCI values, but it is also reliant on other factors. Whereas the ISNCSCI values relate more closely to the neurological severity of the injury, the fusion length mediates the cervical range of motion and, therefore, has critical functionality in daily tasks that involve movements of the head, such as self-care and mobility. We could independently show that cervical mobility, especially flexion in the frontal plane, as well as rotation of the cervical spine, negatively correlates with fusion length [24]. Both are represented prominently in the SCIM [13,14,15,16]. The lack of multicollinearity between the ISNCSCI and fusion length determined in the multiple linear regression model via VIFs further supports this conclusion. The injury severity alone, just as fusion length alone, does not appropriately predict rehabilitation success. Within our cohort, we could prove that they are both relevant and significant predictors of long-term outcomes and should not be misinterpreted as mediators for each other. 

For rehabilitation to succeed, it is very important to plan and execute a rigorous regimen of therapy, exercise, and empowerment according to the individuals’ barriers. A personalized approach is necessary, and fusion length should therefore be routinely assessed in those with cervical spinal cord injuries by rehabilitation professionals and taken into account in the rehabilitation planning process. Physical therapy especially needs to address the impairment of mobility associated with vertebral fusions so that daily tasks represented within the SCIM can be performed to a better extent in those with longer fusions. To dismiss fusion length as a potential indicator for challenges within the long-wearing rehabilitation of patients suffering from cervical SCI could harm the patient’s road back to an independent life as their impairment could be shown to be underestimated with established instruments, such as the ISNCSCI and SCIM.

Our study is somewhat limited due to its monocentric design and may be influenced by institutional bias, but since no other authors have published their research on fusion length and functional outcome regarding SCI yet, we were unable to compare our findings to recent literature on the subject. We hope to encourage more researchers to investigate and publish their findings.

The considerable size of our study population, as well as our carefully selected exclusion criteria, minimized the probability of unique conditions intervening with our results. Uncertainty can be minimized with this method but, of course, cannot be completely dismissed. For example, we did not include the individuals’ genders in our multiple linear regression analysis as the literature does not suggest an effect on rehabilitation [25,26]. We also could not take different surgical approaches into account due to small case numbers. Other authors have suggested that the anterior approach may be the most beneficial [27,28]. It is also by far the most common among our collective. In addition, four participants with fusions, including their atlas or axis, were included. As other studies have shown, loss of function is inevitable while stabilizing these vertebrae [29]. Since only four participants were included, we were unable to assess the influence of this condition. However, a nonstatistical comparison between the four cases and others with similar-sized lengths of fusion did not indicate an additional loss of functionality which could be explained by potential floor effects of the SCIM, as reported by other authors [30].

The results were mathematically limited by the fact that our SCIM II regression analysis presented an adjusted Nagelkerke’s R^2^ of approximately 0.29, which suggested moderate model fitness [31]. With moderate model fitness, it is possible that the correlation between the SCIM II and the other factors was nonlinear, which could not be properly reflected by a multiple linear regression model. However, as a benefit of the method used, it only systematically underestimates the significance of nonlinear correlations. This means that if a nonlinear correlation were present, in reality, the impact would be higher than our conclusions imply.

With this in mind, we could identify the length of fusion, ISNCSCI score at admission and SCIM at admission as significant predictors of the SCIM difference. The length of fusion showed the biggest impact per added unit of the three.

Our findings regarding the neurological level of injury and its insignificance as a predictor could not confirm the results presented by Aidinoff et al., who conducted research using the SCIM III on AIS type A individuals [32]. This insignificance, albeit by a small margin, is most likely due to our inclusion of ISNCSCI scores in our model, as they were heavily dependent on the level of injury. Still, the inclusion of the level of injury was determined to be beneficial as it provided additional model fitness, and multicollinearity could be mathematically disproven.

Age could not be identified as a possible predictor. Ceiling effects are a possible cause but are unlikely since the SCIM values at admission were taken into the analysis, and no relevant ceiling effects in cervically injured individuals were identified with any version of the SCIM by other authors [30,33]. It is more likely that the age at injury between 18 to 50 years old does not significantly influence the functional outcome. Of course, this means that our results cannot be generalized to patients outside of this range without limitations.

The time until admission to the SCI center was not found to be a significant predictor. However, this was by a small margin (*p* = 0.08). It is, therefore, possible that a longer time until admission to a specialized rehabilitation facility does, in fact, negatively influence later functionality, but a significant threshold could not be reached within our population. It is, therefore, possible that a longer time until admission to a specialized rehabilitation facility does, in fact, negatively influence later functionality and should be investigated further.

Considering all the limitations, we conclude that a significant correlation between length of fusion and patient outcome exists independently of previously established predictors of rehabilitation success, such as the ISNCSCI. This knowledge can be used in rehabilitative planning and while determining the potential outcome of patients entering the rehabilitative process.

We also conclude that the causal chain of events leading to this evident correlation remains subject to speculation and is likely influenced by the complexity of injury leading to longer fusion lengths and, therefore, influences the worse outcome of those with longer fusions. Yet, we deem it obligatory to further investigate the negative implications of longer fusions and include the implications of this study in the acute treatment decisions so that potential decisions to favor the immediate surgical success by the performing surgeon do not potentially harm the patients’ rehabilitative success in the long run, if avoidable after a thorough risk assessment. Stability should in no way be compromised in favor of a potentially better long-term outcome, but in those cases where a potential trade-off is possible and in line with reasonable risk, the results of this study should be put in favor of a shorter fusion length.

## Figures and Tables

**Table 1:** Details on the demographics and injury characteristics at admission.

	Group	*n*	Relative
Gender	Male	170	85.4%
	Female	29	14.6%
Level of neurological injury	C0–C1	3	1.5%
	C2	2	1.0%
	C3	42	21.1%
	C4	80	40.2%
	C5	42	21.1%
	C6	12	6.0%
	C7	17	8.5%
	C8	1	0.5%
AIS type	A	99	49.7%
	B	38	19.1%
	C	38	19.1%
	D	23	11.6%
	E	1	0.5%
Cause	Motor vehicle accidents	80	40.2%
	Jumps in shallow waters	57	28.6%
	Fall inflicted injuries	36	18.1%
	Other	26	13.1%

C0–C8 = neurological levels of injury determined via the International Standards for Neurological Classification of Spinal Cord Injury at admission. AIS = American Spinal Injury Association-Classification Impairment Scale

**Table 2:** Length of fusion and frequency of the neurological level of injury.

Length of Fusion	C0–1	C2	C3	C4	C5	C6	C7	C8
2	2	1	14	28	7	2	4	0
3	1	1	17	31	24	8	10	1
4	0	0	5	12	6	1	3	0
5	0	0	2	5	2	1	0	0
6	0	0	1	2	3	0	0	0
7	0	0	1	1	0	0	0	0
8	0	0	2	0	0	0	0	0
9	0	0	0	0	0	0	0	0
10	0	0	0	1	0	0	0	0

Length of fusion = number of vertebrae, C0–C8 = neurological level of injury.

**Table 3:** Upper fusion levels and surgical approaches.

Upper Fusion Level	Number of Patients	Anterior	Combined	Posterior
Occiput	2	0	1	1
Atlas	2	0	1	0
Axis	5	1	2	0
Vertebra 3	35	28	5	2
Vertebra 4	72	60	10	2
Vertebra 5	49	38	10	1
Vertebra 6	32	29	3	0
Vertebra prominens	2	0	1	1

Anterior, combined, posterior = surgical approach for each upper fusion level.

**Table 4:** ISNCSCI values at admission and discharge divided by fusion length.

Length of Fusion		Motor Score T1	Sensory Score T1
2	*n*	58	58
3		93	92
4		27	27
5		10	10
6		6	6
7		2	2
8		2	2
9		0	0
10		1	1
2	Mean ± SD	27.12 ± 23.93	85.97 ± 52.49
3		24.48 ± 22.48	83.01 ± 55.19
4		20.41 ± 17.71	65.78 ± 39.85
5		30.20 ± 20.78	82.50 ± 48.23
6		16.67 ± 14.00	85.00 ± 50.53
7		9.00 ± 8.49	71.50 ± 61.52
8		11.00 ± 15.56	91.00 ± 117.38
9		-	-
10		8.00	12.00

T1 = time of admission, *n* = number of patients, length of fusion = number of involved vertebrae.

**Table 5:** SCIM values at admission and discharge divided by fusion length.

Length of Fusion		SCIM T1	SCIM T2	SCIM T2|T1 Difference
2	n	58	58	58
3		92	93	92
4		27	27	27
5		10	10	10
6		6	6	6
7		2	2	2
8		2	2	2
9		0	0	0
10		1	1	1
2	Mean ± SD	13.83 ± 11.50	49.10 ± 28.21	35.28 ± 25.12
3		14.25 ± 10.79	40.95 ± 24.70	26.21 ± 20.70
4		14.52 ± 13.24	37.93 ± 23.873	23.41 ± 22.10
5		14.20 ± 9.72	41.60 ± 25.94	27.40 ± 20.20
6		8.17 ± 6.59	26.67 ± 8.04	18.50 ± 6.44
7		7.50 ± 3.54	44.00 ± 25.46	36.50 ± 21.92
8		4.50 ± 0.71	10.00 ± 11.31	5.50 ± 10.61
9		-	-	-
10		5.00	22.00	17.00

SCIM = Spinal Cord Independence Measure, T1 = time of admission, T2 = time of discharge, *n* = number of patients, discrepancies in differences can be explained by differing case numbers, length of fusion = number of involved vertebrae.

**Table 6:** Multiple linear regression analysis of the SCIM difference.

Variable	B	SE	*p* value	95% Confidence Interval	
				Lower	Upper
Intercept	13.4	7.6	0.08	−1.8	28.4
SCIM T1	−0.39	0.14	0.01	−0.67	−0.12
Age at injury	0.10	0.15	0.50	−0.19	0.39
Time until admission	−0.16	0.09	0.08	−0.35	0.02
Level of injury	1.9	1.2	0.10	−0.40	4.3
Total ISNCSCI T1	0.17	0.02	<0.01	0.13	0.22
Length of fusion	−2.4	1.1	0.03	−4.6	−0.21
R^2^ = 0.31; adjusted R^2^ = 0.29; F(6, 190) = 14.5, *p* < 0.001					

B = correlation coefficient, SE = standardized error, *p* value = alpha error probability, T1 = time of admission.

## Data Availability

The datasets generated and analyzed during the current study are available from the corresponding author upon reasonable request.
